# Resuscitating the Baby after Shoulder Dystocia

**DOI:** 10.1155/2016/8674167

**Published:** 2016-07-17

**Authors:** Savas Menticoglou, Carol Schneider

**Affiliations:** Division of Maternal-Fetal Medicine, Department of Obstetrics, Gynecology and Reproductive Sciences, University of Manitoba, WR125-735 Notre Dame Avenue, Winnipeg, MB, Canada R3E 0L8

## Abstract

*Background*. To propose hypovolemic shock as a possible explanation for the failure to resuscitate some babies after shoulder dystocia and to suggest a change in clinical practice.* Case Presentation*. Two cases are presented in which severe shoulder dystocia was resolved within five minutes. Both babies were born without a heartbeat. Despite standard resuscitation by expert neonatologists, no heartbeat was obtained until volume resuscitation was started, at 25 minutes in the first case and 11 minutes in the second. After volume resuscitation circulation was restored, there was profound brain damage and the babies died.* Conclusion.* Unsuspected hypovolemic shock may explain some cases of failed resuscitation after shoulder dystocia. This may require a change in clinical practice. Rather than immediately clamping the cord after the baby is delivered, it is proposed that (1) the obstetrician delay cord clamping to allow autotransfusion of the baby from the placenta and (2) the neonatal resuscitators give volume much sooner.

## 1. Introduction 

When shoulder dystocia takes five or more minutes to resolve, the neonate is likely to be depressed and need vigorous resuscitation [1]. We have personal knowledge of two babies that died after there was shoulder dystocia, even though the shoulder dystocia was resolved in less than 5 minutes and the babies had received standard neonatal resuscitation.

## 2. Case  1 

The fetal heart rate tracing had been normal. The second stage lasted less than 1 hour. The head delivered with maternal effort alone and a loop of cord was passed over the baby's neck. Shoulder dystocia was resolved with a combination of McRoberts' maneuver, suprapubic pressure, Rubin's maneuver, and Woods' corkscrew maneuver. The elapsed time from delivery of the head to delivery of the baby was 4.5 minutes.

The cord was immediately clamped and the baby handed to the waiting neonatal team. There was no heart rate or respiratory effort. Resuscitation included positive-pressure ventilation, chest compressions, intubation, and epinephrine. Volume resuscitation began at 25 minutes of age, and the heart rate became detectable after 120 mL of normal saline had been infused.

The birth weight was 4.4 kilograms. The umbilical cord artery pH was 7.16 and the venous pH was 7.27. Following resuscitation, the arterial pH was 6.76. The baby had severe encephalopathy, and brain MRI showed damage to the deep gray structures and brainstem. Care was withdrawn after 1 week of life.

## 3. Case  2 

Labor was induced because of insulin-treated diabetes. There was no concern about the fetal heart rate tracing. The head was delivered by vacuum after three pulls. A nuchal cord was swept over the head. Shoulder dystocia was resolved by McRoberts' positioning, suprapubic pressure, and delivery of the posterior arm. The head-to-body interval was 4 minutes.

The cord was immediately clamped and the neonatology team took over. There were no spontaneous respirations. Positive-pressure ventilation was initiated and intubation was performed for ongoing ventilation. A heartbeat was not audible, and chest compressions were started within the first minute of birth. Ventilation, chest compressions, and epinephrine were continued. At 11 minutes of age, a 60 mL bolus of normal saline was pushed through an umbilical venous catheter. The heartbeat became audible within 1 minute. Another 45 mL of normal saline was administered between 25 and 48 minutes of age.

The birth weight was 4.3 kilograms. The umbilical cord artery pH was 7.05 and the umbilical venous pH was 7.14.

The heart rate was over 100, but the baby remained flaccid with no spontaneous movements, other than spontaneous respirations. Perfusion was poor. Arterial pH at 1 hour of age was 6.92 with a lactate of 20. There was no spontaneous movement, no response to stimuli, and no reflexes. The arterial gases continued to deteriorate, and the baby died at 2.5 hours of age.

## 4. Discussion 

In both of these cases, the fetal heart rate tracings had not shown fetal compromise before the occurrence of the shoulder dystocia. In each case, the shoulder dystocia was resolved in less than 5 minutes. The babies were resuscitated by neonatologists according to the Neonatal Resuscitation Program guidelines. But the babies still died.

This has been described before. In the UK review [2] of 56 cases of babies dying after shoulder dystocia, in 45 of the 56 cases a reasonable estimate of the head-to-body interval could be made. In 21 of the 45 cases (47%), the interval between delivery of the head and the rest of the baby was less than 5 minutes. The authors speculated that perhaps some of the failed resuscitations were caused by preexisting fetal distress before the occurrence of shoulder dystocia or that the head-to-body interval had been underestimated or that compression of the fetal neck during the shoulder dystocia had caused cerebral venous obstruction or excessive vagal stimulation and bradycardia.

In animal experiments [3, 4] where fetuses are subjected to complete anoxia, if the anoxia lasts less than 10 or so minutes and then there is good resuscitation, it is almost always possible to resuscitate the newborn animal without any brain damage. In human instances of obstetrical catastrophes where there is a sudden, acute, severe prolonged bradycardia, perhaps due to a ruptured uterus or placental abruption or cord prolapse, if one can deliver the baby within 10 to 12 minutes of the catastrophe, the result is almost always an undamaged baby [5–8].

One possible explanation for the different outcome in shoulder dystocia was proposed a few years ago [9]. The hypothesis is that, during severe shoulder dystocia, not only is there an acute interruption of oxygen supply to the fetus, but the fetus also becomes hypovolemic. In the first few minutes of shoulder dystocia, the fetal heart is able to continue pumping blood through the higher pressure, less compressible umbilical arteries to the placenta. However, the fetal thorax and/or the umbilical cord is being compressed, and because of the lower pressure in the umbilical vein or because of the increased intrathoracic pressure, blood that has been pumped to the placenta cannot return to the baby's heart. In other words, during some instances of shoulder dystocia, not only is the fetus not receiving oxygen, but the fetus is also losing blood into the placental bed and, thereby, becoming hypovolemic as well. The situation would be analogous to rare cases of neonatal hypovolemic shock reported with tight nuchal cord, where presumably the more easily compressible umbilical vein is occluded but fetal blood is getting pumped to the placenta through the less compressible umbilical arteries [10, 11].

For the obstetrician who has successfully resolved a severe shoulder dystocia, with all its attendant stress and anxiety, the natural response, as soon as the baby is delivered, is to immediately clamp the umbilical cord and hand the baby over to the waiting neonatal resuscitation team. This is the actual recommendation of the Society of Obstetricians and Gynaecologists of Canada, “if the fetus is depressed, then the baby should be handed over for immediate resuscitation… [12].” This is what was done in our cases. If the hypothesis that babies born after shoulder dystocia may also be in hypovolemic shock is correct, then the correct approach, instead of immediately clamping the umbilical cord, would be to leave the umbilical cord unclamped for a minute or so to allow blood to return from the placenta through the now noncompressed umbilical vein and noncompressed thorax to the baby's heart and circulation. As it is, the first 60 seconds of the standard neonatal resuscitation protocol are taken up with drying and stimulation and positive-pressure ventilation with a bag and mask. This first minute of resuscitation can be done at the mother's bed. Instead of clamping the cord immediately and taking the baby to the resuscitation warmer, the resuscitation team can come to the mother's bed and begin the resuscitation steps with the baby still attached to the umbilical cord between the mother's legs. Alternatively, there are commercially available neonatal resuscitation carts that can be brought to the mother's bedside to begin the resuscitation.

If the hypothesis of hypovolemic shock is correct, then a change may be needed in the neonatal resuscitation protocols. Current neonatal resuscitation guidelines [13] actually discourage volume expansion:

“Volume expanders should not be routinely given during resuscitation in the absence of a history or indirect evidence of acute blood loss. Giving a large volume load to a baby whose myocardial function is already compromised by hypoxia can decrease cardiac output and further compromise the newborn…”

“Indications for volume expansion during resuscitation include:

(i) Baby is not responding to resuscitation.

AND

(ii) Baby appears in shock (pale color, weak pulses, persistently low heart rate, no improvement in circulatory status despite resuscitation efforts).

OR

(iii) There is a history of a condition associated with fetal blood loss (e.g.: extensive vaginal bleeding, placenta previa, twin-to-twin transfusion, etc.).”

The neonatal resuscitation algorithm comprises assessment and initial steps; positive-pressure ventilation; positive-pressure ventilation and chest compressions; positive-pressure ventilation, chest compressions, and epinephrine and endotracheal intubation; and, finally, umbilical venous catheterization for volume replacement if there has not been a good response to the aforementioned steps. All this generally means that at least 10 minutes will elapse before any volume will be given. And then the suggested volume to be given, 10 mL/kg of saline over 5 to 10 minutes, will probably be inadequate if the fetus has lost a significant amount of blood.

Of course, the hypothesis that some babies who have been delivered after shoulder dystocia are in hypovolemic shock is still a hypothesis (the article was indeed published in a journal called Medical Hypotheses), but the circumstances of our two cases lend support to this hypothesis. In the first case, there was no detectable heartbeat until volume resuscitation was begun at 25 minutes of age and 120 mL of normal saline was infused. In the second case, when there was still no detectable heart rate after 11 minutes of standard resuscitation, 60 mL of normal saline was pushed through an umbilical venous catheter and a heart rate was detectable within a minute afterwards. In both cases, however, it was too late.

What are the implications for management of severe shoulder dystocia?

For the obstetrician, once the shoulder dystocia has been resolved and the baby is delivered, it would be to restrain the urge to immediately clamp the umbilical cord but, instead, keep the baby between the mother's legs for a minute or so, to let the baby be autotransfused from the placenta, and have the neonatal resuscitation team come and do the initial resuscitation steps at the mother's bed. Alternatively, the umbilical cord can be milked with some benefit [14].

For the neonatal resuscitators, it would be to consider volume expansion much earlier in the resuscitation algorithm and put in an umbilical venous catheter and give volume sooner if there is no detectable heartbeat after 1 or 2 minutes. If the placenta has been delivered, one could consider taking blood from the vessels on the fetal surface of the placenta and using that to transfuse the baby with its own blood (which would also contain many stem cells that could assist the baby to repair any damage).
